# Cognitive Benefits of Social Dancing and Walking in Old Age: The Dancing Mind Randomized Controlled Trial

**DOI:** 10.3389/fnagi.2016.00026

**Published:** 2016-02-22

**Authors:** Dafna Merom, Anne Grunseit, Ranmalee Eramudugolla, Barbara Jefferis, Jade Mcneill, Kaarin J. Anstey

**Affiliations:** ^1^School of Science and Health, Western Sydney University, Penrith, NSW, Australia; ^2^Prevention Research Collaboration, School of Public Health, University of Sydney, Sydney, NSW, Australia; ^3^Centre for Research on Aging, Health and Wellbeing, The Australian National University, Canberra, ACT, Australia; ^4^Department of Primary Care and Population Health, University College London, London, UK; ^5^Early Start Research Institute, School of Education, University of Wollongong, Wollongong, NSW, Australia

**Keywords:** executive functions, dance, walking, physical function, physical activity

## Abstract

**Background:**

A physically active lifestyle has the potential to prevent cognitive decline and dementia, yet the optimal type of physical activity/exercise remains unclear. Dance is of special interest as it complex sensorimotor rhythmic activity with additional cognitive, social, and affective dimensions.

**Objectives:**

To determine whether dance benefits executive function more than walking, an activity that is simple and functional.

**Methods:**

Two-arm randomized controlled trial among community-dwelling older adults. The intervention group received 1 h of ballroom dancing twice weekly over 8 months (~69 sessions) in local community dance studios. The control group received a combination of a home walking program with a pedometer and optional biweekly group-based walking in local community park to facilitate socialization.

**Main outcomes:**

Executive function tests: processing speed and task shift by the Trail Making Tests, response inhibition by the Stroop Color-Word Test, working memory by the Digit Span Backwards test, immediate and delayed verbal recall by the Rey Auditory Verbal Learning Test, and visuospatial recall by the Brief Visuospatial Memory Test (BVST).

**Results:**

One hundred and fifteen adults (mean 69.5 years, SD 6.4) completed baseline and delayed baseline (3 weeks apart) before being randomized to either dance (*n* = 60) or walking (*n* = 55). Of those randomized, 79 (68%) completed the follow-up measurements (32 weeks from baseline). In the dance group only, “non-completers” had significantly lower baseline scores on all executive function tests than those who completed the full program. Intention-to-treat analyses showed no group effect. In a random effects model including participants who completed all measurements, adjusted for baseline score and covariates (age, education, estimated verbal intelligence, and community), a between-group effect in favor of dance was noted only for BVST total learning (Cohen’s *D* Effect size 0.29, *p* = 0.07) and delayed recall (Cohen’s *D* Effect size = 0.34, *p* = *0.06*).

**Conclusion:**

The superior potential of dance over walking on executive functions of cognitively healthy and active older adults was not supported. Dance improved one of the cognitive domains (spatial memory) important for learning dance. Controlled trials targeting inactive older adults and of a higher dose may produce stronger effects, particularly for novice dancers.

**Trial registration:**

Australian and New Zealand Clinical Trials Register (ACTRN12613000782730).

## Background

Cognitive decline has emerged as one of the greatest health threats in old age, with nearly 50% of adults aged 85 or over suffering from dementia (Prince et al., 2013). It is increasingly recognized that dementia may not be an inevitable outcome of aging (Jorm, 2002; Haan and Wallace, 2004). Identification of effective interventions that delay the onset of the disease or slow its progression, even if modestly, hold the promise of substantially reducing the national and individual burden of dementia (Jorm, 2002).

Consistent evidence from population-based cohort studies suggests that a physically active lifestyle in late life can attenuate cognitive decline and reduce the risk of developing dementia (Fratiglioni et al., 2004; Hamer and Chida, 2009; Blondell et al., 2014). Randomized control trials (RCTs) also demonstrate improved cognitive function in older healthy adults on exercise regimens (Colcombe and Kramer, 2003; Colcombe et al., 2004; Angevaren et al., 2008; Brown et al., 2009). The concept that has led intervention research in this area is that cognitive benefits are derived through improved fitness dimensions, such as cardio-respiratory fitness or muscle training. Recently, Voelcker-Rehage et al. (2011) and others demonstrated increase in functional plasticity following coordinative training (Liu-Ambrose et al., 2008), which led to the conclusion that each fitness domain may affect the brain differently (Voelcker-Rehage and Neimann, 2013). Focusing only on measures of fitness to assess the impact of exercise on cognition is not sensitive to the fact that physical activities vary considerably in the degree of sensorimotor complexity, cognitive demand, and social interaction that they entail. These factors may significantly modify the effects of physical activity on the aging brain regardless of changes in fitness. For example, in the field of movement science, activities are classified according to skill progression. As opposed to simple motor skills, which are more predictable, involving repetitive or less variable goal-directed movement (e.g., running or throwing), complex motor skills incorporate significantly higher levels of intricately (organization/components) coordinated body movements requiring learning and practice. Complex motor skills cannot be mastered in a single practice session as they involve unpredictable and changing environments where the person and/or object are in motion, such as ball sports, dance, and martial arts (Gentile, 1972; Kraft et al., 2015).

Physical, mental, and social activities are often conducted independently, but there may be synergetic benefits when delivered in combination. For example, the combination of physical (cycling on a stationary bike, which is a simple motor skill) and cognitive components (using a video-game during the cycling) showed greater benefits than cycling in isolation (Andreson-Hanley et al., 2012). Likewise, older participants in Tai-Chi classes (an activity classified as complex or “multi-dimensional” because it involves balance and strength as well as cognitive control of movement) (Wayne and Kaptchuk, 2008) experienced greater improvements in selected cognitive measures than participants in a traditional Western exercise class (Taylor-Piliae et al., 2010).

Dance has been highlighted as a potentially superior activity for maintaining or improving cognitive ability (Brown et al., 2006). Dancing for humans has been equated to the effect on neurogenesis of animals living in an enriched environment (Kattenstroth et al., 2010). Cognitively, dance requires learning of complex motor sequences, procedural memory, attention, visuomotor integration, synchronization in space and time (rhythm movements), and emotional expression (Brown et al., 2006). In recent years, neuroscientists have used dance as a model for studying neural processes implicated in the execution, expression, and observation of skilled movement (Bläsing et al., 2012). However, this research has been devoted to comparison between young professional dancers and non-dancers but some processes identified may apply to an older population. In addition, dance is fundamentally a social activity that has been shown to promote social engagement in older people (Keyani et al., 2005), which is in turn associated with better cognitive outcomes in longitudinal studies (Fratiglioni et al., 2004). In the only longitudinal study where leisure-time activity-specific benefits were compared, frequent dancing (≥3 times/week) was associated with lower risk of dementia [hazard ratio (HR) of 0.24 CI: 0.06–0.99], similar to playing a musical instrument. By contrast, the association with walking was weaker and of only marginal significance (HR = 0.67 CI: 0.45–1.05) (Verghese et al., 2003). However, observational studies are subject to numerous biases, in particular, “reverse causation” where participation in an activity may be self-selected based on pre-existing or unmeasured cognitive, physiological, and behavioral abilities.

To date, six experimental studies with older participants have examined improvements in cognitive functions in response to any type of dance interventions (Alpert et al., 2009; Coubard et al., 2011; Kim et al., 2011; Kimura and Hozumi, 2012; Kattenstroth et al., 2013; Hackney et al., 2015) indicating conflicting results. However, most studies employed weak designs (i.e., pre–post evaluation or non-equivalent controls and involved small samples <50) (Alpert et al., 2009; Coubard et al., 2011; Kim et al., 2011; Kattenstroth et al., 2013; Hackney et al., 2015), and therefore fall well short of the methodological quality required to test the efficacy of dance as a cognitive intervention. The only randomized controlled trial, to date, was limited to the immediate effect of free style aerobic dancing lasting a total of 40 min (Kimura and Hozumi, 2012); hence, the long-term cognitive effects of exposure to dance remains unknown.

We therefore conducted the first randomized controlled trial of a dance-based intervention, which aimed to compare (i) changes in age-sensitive cognitive domains between two groups of older adults (60+) randomly assigned to either ballroom dancing (representing a complex multi-dimensional physical activity) or walking (representing the simplest, functional, and most accessible physical activity in old age); (ii) changes in exercise capacity and sensorimotor capabilities between the two groups, and whether these changes were associated with cognitive changes.

## Materials and Methods

A pragmatic randomized controlled trial was conducted from April 2013 to September 2014. The study protocol was approved by the Human Research Ethics Committee (ref: 9987) University of Western Sydney. The trial was registered a day after the first participant was enrolled (retrospective registration) with the Australian and New Zealand Clinical Trials Register (ACTRN12613000782730).

## Setting and Participants

Participants were recruited in a staggered manner from five suburbs around Sydney. The selection of the suburbs was based on (i) availability of an accessible dance studio (i.e., walking – distance from public transport); (ii) dance studio space at least 10 m long; (iii) owner is a ballroom teacher; (iv) owner agreed to volunteer the studio space for the measurements without cost. After publicity in community newspapers and information sessions in various seniors’ community clubs, interested participants were invited to call the research center for a short screening interview to determine eligibility and get more information.

Eligible participants had to be ≥60 years of age and be able to walk unaided for at least 50 m. Participants also had to have GP clearance if they suffered from an unstable or chronic condition limiting their participation in regular exercise (e.g., unstable ischemic heart condition, hypertension, and debilitating arthritis). Participants were excluded if they had significant cognitive impairment determined by <21 points on the Telephone Interview of Cognitive Status (TICS), which is the telephone modification of the Mini-Mental Cognitive Status Examination (MMSE; equivalent to 24) (Welsh et al., 1993).

### Dance Group

A collection of ballroom dances, Rock and Roll, Foxtrot, Waltz, and some Latin (Salsa and Rumba) were delivered twice weekly for 1 h over 8 months (~69 h). The program was progressive in terms of motor complexity (e.g., Foxtrot: Basic steps, Promenade, Rock Turn, and Sway). The program was standardized across five studios and five instructors in a 4-h workshop at the start of the study. In the workshop, teachers received the program resources (CDs for music and workbook) and were introduced to the potential difficulties of delivering dance classes to an older population.

### Walking Group

The “control” group received previously published home-based self-help walking program with a pedometer (Merom et al., 2007) and were asked to walk the equivalent in terms of hours per week as the dance intervention, over 8 months. They were also offered group meetings once every fortnight in a designated community park to provide opportunities to socialize. In between the group sessions, participants were encouraged to walk in their residential areas alone or with others and to reach a step target. A progressive goal was provided every 3 months but adherence to the goals was not tracked, in line with the Step-by-Step program effectiveness trial (Merom et al., 2007).

### Data Collection Procedures and Measurement Schedule

Eligible participants were invited to a 1-h baseline physical and cognitive assessment in the community dance studio and to a second baseline, 3 weeks later, to repeat only the cognitive tests. The delayed baseline aimed to estimate the effect of spontaneous learning just from repeated measurement. The assessments included a self-complete questionnaire including self-report physical activity using the validated CHAMPS questionnaire (Giles and Marshall, 2009), objective assessment of physical activity using Actigraph GTX on a sub-sample, past experience of dancing, existence of chronic conditions and socio-demographics.

At the end of 8 months, a follow-up assessment was scheduled to repeat all baseline measurements. One research assistant (Jade Mcneill) was trained in all the cognitive and physical measurements and administered the tests at all-time points.

### Randomization and Blinding

An *a priori* plan for random assignment of 126 individuals (see Sample Size and Statistical Analysis) was prepared by the study statistician (Anne Grunseit) using computer software. A letter informing the allocation was sent to participants by the research assistant (Jade Mcneill) after the “delayed baseline.” Hence, participants and the research assistant were blind to allocation during the two baseline assessments but not at follow-up.

### Main Cognitive Outcome Measures

#### Executive Function

Processing speed and Task switching/cognitive flexibility were measured by the Trail Making Tests (TMT) Parts A and B (Tombaugh, 2004). Part A measures processing speed and involves participants connecting consecutive numbers (e.g., 1–2–3). Part B provides a measure of “task shifting” and involves participants connecting alternate letters and numbers (e.g., 1–A–2–B). The difference in time between the two parts (B − A) was calculated to isolate the executive component of these tests indicating cognitive flexibility.

Response inhibition was measured by the Stroop Color-Word Test (SCWT), which specifically measures the ability to suppress a pre-potent response. Participants were first shown a page with color dots and were asked to name the color. Second, participants were shown color names printed in non-matching ink (i.e., the word blue is printed in red). In this part, they were asked to name the color of the ink in which each word was printed while suppressing the tendency to read the word itself. The difference in time between the two parts (second test – first) isolates the executive component of response inhibition.

Working memory was measured by the Digits Span Backwards (DSB). Seven pairs of random number sequences were read to participants. The sequence began with three digits and increased by one each trial, up to a maximum of nine digits. The maximum length of digit sequence correctly repeated in reverse was recorded.

#### Learning and Memory

Verbal immediate and delayed memory and learning, including retroactive inhibition, and retention: this was measured by the Rey Auditory Verbal Learning Test (RAVLT). Participants were asked to recall from a list of 15 words (List A) as many words as they could (immediate recall). The same list was read five times and the sum of correct words recalled over all trials was recorded. A second distracter list of 15 words was read (List B) after which in the sixth trial participants were asked to retrieve as many words from list A. Delayed recall was measured after 20 min, during which other physical tasks were administered, when participants were asked again to recall the 15 words from list A.

Visuospatial immediate and delayed memory was measured using the Brief Visuospatial Memory Test (BVMT). Participants were shown six Geometric visual designs in a 2 × 3 matrix. The stimulus was presented for 10 s, after which it was removed from view. BVMT1 is a measure of short-term retention of visual information. BVMT total score is a measure of acquisition of figural/spatial information generated by summing the scores from three trials. The BVMT4-delayed recall was done 25 min later.

### Physical Measures

The 6-minute walking test (6MWT) was conducted as a proxy measure for exercise capacity (Enright, 2003). The test is easier to administer and better tolerated by older adults than other measures of cardio-respiratory capacity. Functional mobility was measured by time to complete five-chair rises and by gait speed, which was calculated from the fastest time to walk 6 m from two trials.

### Covariates

#### Estimate of Verbal Intelligence

Word Knowledge was measured by the *Spot the Word test*. Participants were shown pairs of words in which one was a real word and one “invented” word. The number of correctly identified real words out of a total of 60 was recorded. Social networking was recorded using Lubben et al. (2006) abbreviated validated tool.

#### Socio-Demographic Details

Socio-demographic details include age, gender, the highest level of education achieved, work status, English as a second language.

### Sample Size and Statistical Analysis

Based on systematic reviews, the pooled effect size of aerobic interventions of 6 months was 0.26 for executive functions tests in comparison to no treatment control (Angevaren et al., 2008). We hypothesized that multi-dimensional aerobic physical activity would result in medium effect size (0.5) at 8 months over a no treatment control group, as we assumed that an unsupervised walking intervention would have minimal cognitive effect, as previously indicated (Uffelen van et al., 2008). Power calculations (Faul et al., 2007) based on this effect size and an attrition rate of 18%, yield a sample size of 126 (power = 0.80, α = 0.05).

**Figure 1:**
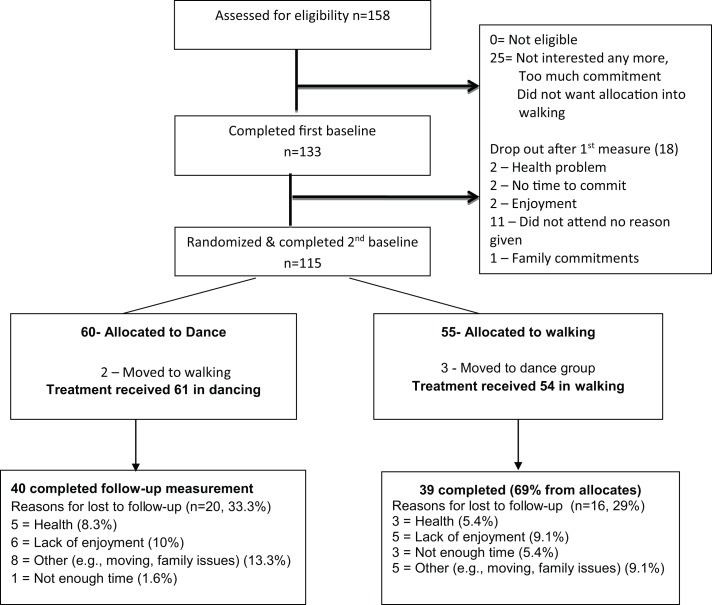
**Participant flow by study arm**.

The differences in change scores between the two groups were initially tested by the intention-to-treat (ITT) principle, where missing data were imputed from the last known observation. However, because five participants swapped groups despite accepting the rule of randomization (see Figure 1) most of the reported analyses refer to the “treatment received” outcomes for those with full data at three time points, hereafter “completer.” The changes in cognitive scores were evaluated first without adjustment, using paired *t*-tests and within- and between-groups differences using Cohen’s *D* effect size (Dunlap et al., 1996) Further analyses were conducted to allow for simultaneous comparisons across all three time points with the inclusion of covariates using linear mixed random models for continuous outcomes. The effect of group on change in cognitive outcome was tested by including a group by time interaction term.

## Results

Figure 1 presents the recruitment process and the flow of participants during the trial.

All the 158 participants who called the center and were screened were cognitively and physically eligible but only 133 participants (84%) agreed to participate. Reasons for declining to take part were associated with difficulty with committing to days/time, or not accepting the randomization rule. Eighteen participants (13%) withdrew before the delayed baseline and randomization, for reasons unknown, leaving 115 participants who were randomized (60 to dance and 55 to walk). Retention to study end was slightly lower in the dance group (66%) than the walking group (69%) due to the greater number of participants lost to follow-up in that group.

The baseline characteristics of the study participants by random allocation as well as by “treatment received” for completers are presented in Table 1. At baseline and after randomization, the groups were well balanced in terms of socio-demographic characteristics, health, and cognitive status. Self-report leisure-time physical activity indicated that one-third of study participants already exercised 2.5 h/week or more, including 12% in each group who danced in the past month (data not shown). Objective monitoring using accelerometer also indicated a higher proportion of active participants allocated to walking (58.1%) compared to dance (36.7%) met the health recommendation of  ≥30 min of moderate to vigorous physical activity daily (*p* = 0.04). There was a tendency for the dance group to have a higher score on the measure of verbal intelligence (Spot the Word Test). The groups were well balanced in terms of the main cognitive outcome measures with the exception of the BVMT total learning and gait speed, which showed significantly better scores among dance participants.

**Table 1:** **Baseline characteristics of study participants and baseline scores of outcome measures by group allocation at randomization and by treatment received of “completers**.”

Characteristic	Group allocation as randomized		*p*-Value^a^	Completers by treatment received		*p*-Value^a^
	Dance *n* = 60	Walk *n* = 55		Dance *n* = 40	Walk *n* = 39	
**Gender (%)**			0.40			0.96
Female	73.3	80.0		85.0	84.6	
**Age (%)**			0.45			0.58
60–69 years	48.3	60.0		50.0	61.5	
70–74 years	25.0	20.0		25.0	18.0	
75+ years	26.7	20.0		25.0	20.5	
**Highest education (%)**			0.69			0.46
Primary/high school	45.0	52.7		37.5	48.7	
TAFE^/^apprenticeship	21.7	20.0		27.5	28.2	
University	33.3	27.3		35.0	23.1	
**Work status (%)**			0.13			0.09
Not retired	10.0	20.0		7.5	20.5	
Retired	90.0	80.0		92.5	79.5	
**Self-rated health (%)**						
Excellent/very good	70.2	53.7	0.20	69.0	54.7	0.30
Good	26.3	40.7		27.6	39.6	
Fair/poor	3.5	5.6		3.5	5.7	
**Physical activity/dance (%)**						
Exercising >2.5 h/week	38.3	32.7	0.54	45.0	33.3	0.29
MVPA ≥30 min/day	36.7	58.1	0.04	50.0	58.6	0.49
Past experience in dancing	45.0	46.3	0.89	40.0	46.1	0.58
**Cognitive measures (means)**						
TICS (number correct, 0–39)	28.1	27.9	0.73	28.2	27.8	0.62
Spot the word (14–60)	50.3	48.4	0.18	50.2	48.6	0.26
RAVLT-immediate recall (0–15)	5.8	6.0	0.65	6.4	6.0	0.26
RAVLT-delayed recall (0–15)	8.8	9.3	0.40	9.3	9.2	0.35
TMT-A (s)	40.8	42.2	0.78	37.4	37.8	0.84
TMT-B (s)	96.5	106.0	0.28	88.5	100.3	0.91
Digits backwards	6.5	6.4	0.74	6.8	6.6	0.16
BVMT-immediate recall	4.3	3.5	0.07	4.2	3.3	0.67
BVMT total learning	19.9	17.2	0.04	20.1	17.4	0.08
BVMT-delayed recall	8.3	7.6	0.18	8.4	7.7	0.07
Stroop trial 1 (s)	14.7	15.5	0.48	14.1	14.3	0.28
Stroop trial 2 (s)	36.2	34.6	0.47	35.2	34.3	0.81
**Physical function (means)**						
6MWT (m)	496.8	489.1	0.68	496.6	489.1	0.69
Five-chair stands (s)	10.6	11.2	0.30	11.1	11.1	0.95
Gait speed (m/s)	1.4	1.3	0.05	1.4	1.3	0.16
**Social network (score)**	22.8	22.5	0.73	23.4	22.9	0.69

*^a^*p*-Value for between-groups differences*.

*TAFE, TAFE is a vocational college; MVPA, moderate to vigorous activity measured by accelerometer, *n* = 93; TICS, Telephone Interview Cognitive Status; RAVLT, Rey Auditory Verbal Learning Task; BVMT, Brief Visuospatial Memory Test; 6MWT, 6-minute walk test*.

The “treatment received” and completers groups (Table 1) did not differ significantly. However, the dance group had a greater loss of men (*p* = 0.002) and of participants in the lowest education category (*p* = 0.065), when compared to the walking group. Furthermore, significant differential attrition by cognitive scores at baseline was noted for the dance groups (Table 2) where those with the poorest baseline performance on immediate and delayed verbal recall (RAVLT), executive function (TMTA, TMTB), and working memory (DB) left the study. By contrast, with the exception of TMT part A, completers in the walking group were not different from non-completers.

**Table 2:** **Comparison of baseline scores between study completers and non-completers stratified by “treatment received” groups**.

	Dance			Walk		
	Non-completers (*n* = 20)	Completer (*n* = 40)	*p*	Non-completers (**n* = 16)*	Completer (*n* = 39)	*p*
RAVLT1-immediate recall	4.2	6.45	0.000	5.9	6.05	0.378
RAVLT7-delayed recall	7.5	9.32	0.049	9.6	9.18	0.690
TMTA total time (s)	51.2	37.4	0.051	48.4	37.8	0.032
TMTB total time (s)^a^	117.6	88.5	0.003	113.9	100	0.200
TMTB–TMTA difference (s)^a^	76.4	51.1	0.002	65.5	62.5	0.416
Digits backwards	5.9	6.8	0.048	5.9	6.59	0.154
BVMT1-immediate recall	4.6	4.22	0.713	3.8	3.31	0.763
BVMT total learning	19.1	20.1	0.313	16.8	17.4	0.370
BVMT4-delayed recall	7.7	8.41	0.198	7.5	7.72	0.422
Stroop (trial 3–trial 1, s)	18.7	21.1	0.244	21.4	20	0.638

*^a^Bigger score means poorer performance*.

Adherence to dance intervention among completers was 78% and was not monitored for the walking group as this was primarily a home-based walking program.

### Changes in Cognitive Measures

Intention-to-treat analysis (Table 3) showed significant improvements from baseline to delayed baseline on some tests in both groups, indicating a learning effect without intervention. Further within-group improvements from delayed baseline to study end were small with the highest effect size documented being 0.20 for the BVMT immediate and total learning among walkers, followed by RAVLT tests and Digit backwards, with effect sizes of 0.15 and 0.14, respectively, among dancers.

**Table 3:** **Unadjusted scores for cognitive tests at baseline, delayed baseline, and follow-up, and within-group effects between delayed baseline and follow-up for 115 participants completing delayed baseline**.

	Allocation group dance (*n* = 60)			Cohen’s *D*	Allocation group walking (*n* = 55)			Cohen’s ***D***
	Baseline	Delayed baseline	Follow-up^†^,*		Baseline	Delayed baseline	Follow-up^†^,*
RAVLT1-immediate recall	5.80	6.25	6.61	0.15	5.98	6.05	6.29	0.11
RAVLT7-delayed recall	8.77	8.95	9.02	0.02	9.31	9.11	8.82	0.08
TMTA	40.8	37.8	3.83	0.02	42.2	41.3	41.7	0.02
TMTB total time	96.5	94.3	99.2	0.08	106	107	111	0.05
TMTB-TMTA difference	59.26	56.55*	60.89	0.09	63.59	66.02	69.26	0.06
Digits backwards	6.5	6.42	6.1	0.14	6.36	6.56	6.58	0.01
BVMT1-immediate recall	4.3	5.32*	5.23	0.05	3.5	5**	4.48	0.20
BVMT total learning	19.9	21.3	21.2	0.02	17.2	20.8**	19.5	0.20
BVMT4-delayed recall	8.31	8.1	8.48	0.10	7.56	8.18	7.97	0.07
Stroop 1 (s)	14.7	14.3*	14.1	0.04	15.5	14.4*	14.7	0.04
Stroop 2 (s)	36.2	32.6**	32.7	0.01	35.6	32.7**	33.7	0.08

*T2 vs. T3 one-tailed test for improvement *p* < 0.05 **p < 0.01.

*^†^Using last value carried forward for missing data*.

Completers’ analysis following “treatment received” (Table 4)allocation revealed a similar pattern to ITT analysis; the most improvement occurred between baseline and delayed baseline, and further within-group improvements were small and non-significant for all tests. Unadjusted between-group effect sizes >0.25 (data not shown) were noted for Working memory (DB) in favor of the walking group, and for visuospatial learning (BVMT), visuospatial delayed recall (BVMT4), and executive function response inhibition (Stroop) in favor of the dance group. After adjustment for the differences in baseline scores, time effect and covariates (age, education, pre-morbid intelligence and community), a near-significant effect of dance over walking was noted for visuospatial learning *p* = 0.065, including visuospatial delayed recall (*p* = 0.07).

**Table 4:** **Scores for cognitive tests at baseline (T1), delayed baseline (T2) and follow-up (T3) for 79 participants completing all measurements analyzed as “treatment received” and the adjusted intervention effect using Generalized Linear Model with random effects**.

	Allocation group dance (*n* = 40)			*p*-Value*	Allocation group walk (*n* = 39)			*p*-Value*	Between-groups Cohen’s *D*^†^	*p*-Value group × time interaction^‡^
					
	Baseline	Delayed baseline	Study end		Baseline	Delayed baseline	Study end			
RAVLT1-immediate recall	6.45	6.45	6.83	0.144	6.05	6.05	6.51	0.114	−0.038	0.978
RAVLT7-delayed recall	9.32	9.2	9.15	0.546	9.18	9.15	8.9	0.691	0.070	0.937
TMTA total time (s)	37.4	35.0	36.2	0.696	37.8	36.3	36.2	0.562	0.068	0.934
TMTB total time (s)	88.5	90.2	96.6	0.808	100	92.3	98.5	0.831	0.003	0.534
TMT difference (s)	51.1	55.2	60.4	0.215	62.5	56	61.9	0.160	−0.019	0.471
Digits backwards	6.80	6.67	6.17	0.953	6.59	6.59	6.64	0.422	−0.319	0.162
BVMT1-immediate recall	4.22^a^	5.36	5.22	0.674	3.31	5.08	4.36	0.980	0.235	0.446
BVMT total learning	20.1^a^	21.3	21.3	0.614	17.4	21.4	19.5	0.994	0.291	0.070
BVMT4-delayed recall	8.41	8.03	8.72	0.121	7.72	8.28	8.56	0.818	0.343	0.065
Stroop trial 1 (s)	14.1	13.5	13.1	0.108	14.3	13.6	14.1	0.839	−0.352	0.304
Stroop trial 2 (s)	35.2	31.2	31.6	0.669	34.3	31.7	33	0.831	0.307	0.496
Stroop difference (s)	21.1	17.7	18.5	0.170	20.0	18.1	19	0.251	−0.017	0.685

*For most measures, except TMT and Stroop, higher score at follow up indicates improvement. Therefore a positive intervention effect would result in a positive sign for between group difference with the exception of TMT and Stroop test where negative signs indicate positive intervention effect*.

*T3 vs. T2 one-tailed within-groups test for improvement.

*^†^Test of T3–T2 between-groups difference*.

*^‡^Derived from group (treatment received – walk coded 0, Dance coded 1) by time (baseline, delayed baseline, T3) interaction term in random effects model adjusting for age, education, Spot the word (tertiles), and community with person as the random effect*.

*^a^Test treatment received by cognitive measure at T1 showed Dance superior to Walk*.

### Changes in Secondary Outcomes

No significant between-group effects were documented for any of the physical tests or for social networking (Table 5).

**Table 5:** **Change from baseline (T1) to follow-up (T3) in physical function tests and social network and the adjusted intervention effect using generalized linear model with random effect**.

	Dance (*n* = 40)		Walk (*n* = 39)		Between-group difference^†^
	Baseline	Study end	Baseline	Study end	*p*-Value
Sit–stand test (s)^a^	11.1	11.7	11.1	11.2	0.559
Six-minute walking test (m)^b^	487	494	490	485	0.354
Gait speed (m/s)^b^	1.36	1.33	1.29	1.30	0.582
Social Network (total range 0–30)^b^	23.4	23.3	22.9	23.9	0.469
family subscale (range 0–15)	11.4	11.4	11.5	11.7	0.422
friend subscale (range 0–15)	11.9	11.9	11.4	11.5	0.668

*^†^Test of group difference at T3 adjusted for gender, age, self-rated health status, and baseline test value*.

*^a^Lower score is better*.

*^b^Higher score is better*.

We further examined whether changes in physical tests correlated with changes in the cognitive tests (Table 6); all correlations were in the expected direction in general but the partial correlations (accounting for the age effect) were low. The highest correlation (0.27) was between changes in Stroop Part 1 (color-naming) and the five-chair rises, which is an indirect measure of functional balance.

**Table 6:** **Partial correlations between change in physical and cognitive outcome**.

	Sit–stand test	Gait speed	Six-minute walking
RAVLT1-immediate recall	−0.1209	0.1078	0.1364
RAVLT7-delayed recall	−0.0548	0.0294	−0.1053
TMTA total time (s)	−0.1175	0.0814	−0.122
TMTB total time (s)	−0.1172	0.0338	−0.1751
TMTB-TMTA difference (s)	−0.0955	0.0149	−0.1572
Digits backwards	0.0105	−0.0908	−0.0542
BVMT1-immediate recall	0.072	−0.0407	0.0146
BVMT total learning	0.0896	0.032	0.0532
BVMT4-delayed recall	0.0548	0.0337	−0.067
Stroop trial 1 (s)	−0.2721*	0.0439	−0.1639
Stroop trial 2(s)	0.0384	−0.1859	0.0796
Stroop difference (s)	0.111	−0.1933	0.122

*Statistically significant at *p* < 0.05.

## Discussion

This RCT did not support the hypothesis that multi-dimensional physical activity, such as dance, elicits greater changes in executive function, learning, and memory than a functionally simple motor activity such as walking among cognitively healthy active older adults. The results did suggest that participation in dance may improve visuospatial learning and memory to a greater extent than walking. There were small and non-significant improvements as a result of both interventions, and few significant learning effects were observed just from repeated measurements.

### Interpretation of Results in the Context of Other Research

There are several explanations for the lack of significant effects in either intervention. First, our study participants appeared to be highly active at baseline, particularly the walking group participants, who took 6,162 steps/day on average. A systematic review of six studies investigating the effects of walking interventions on executive functions of cognitively healthy older adults (Scherder et al., 2014) suggests that physical activity levels of participants at baseline matters; the effect sizes of the walking interventions on set shifting and inhibition tests, combined, ranged from 0.17 to 0.48 (pooled effect size was 0.36). Studies reporting large effect, for example, Hiyama et al. (2012) involved participants whose initial level of activity averaged at 4,453 steps/day; the effect on task shifting (TMT difference) was 0.40. By contrast, Oken et al. (2006) included active people in their study, excluding only those doing 3.5 h/week or more of aerobic exercise and no significant treatment effect for walking compared to non-exercise control was found. Engaging older adults who are generally fit and active may leave little room for improvements if, by consequence, the brain already exhibits efficient processing, similar to younger adults (Hollman et al., 2007).

Another explanation could be the nature of intervention, which lacked sufficient physical and mental challenges particularly given such a physically competent group. Previous studies on the effects of aerobic exercise on cognitive plasticity not only recruited inactive populations but also employed a training protocol of high intensity (70% of maximum VO_2_ capacity of each individual) and higher dosage (3 days a week of training) (Colcombe and Kramer, 2003; Colcombe et al., 2006). Furthermore, half of our group were not novice dancers; of those, 12% reported some sort of dancing in the preceding 4 weeks. Most previous dance-based interventions included only novice participants. In the present trial, there were no improvements in physical function tests in both groups, probably due to a ceiling effect. For example, half of our sample completed five-chair rises in <11 s and another 40% performed at the second highest level (11.2–13.6 s) and the mean 6MWT walking test distance was 496.8 m. By comparison, after a Thai dance intervention, the chair rise and 6MWT improved in a sample with poorer baseline functioning (12.9 s for chair rises and 360 m for 6MWT) (Lanyacharoen et al., 2013). In another study with a baseline mean distance of 415 m in 6MWT, after 6 months Latin dance program, the test improved to 499 m, which is very near to the starting point of our sample (Mangeri et al., 2014). The small non-significant changes in physical function may also explain the low correlations documented here with the changes in cognitive tests. Low but significant correlations were also reported for LIFE-P exercise intervention (Williamson et al., 2009), but the functional status of the participants was very low and participants in that trial were much older. However, those with the poorest performance on physical function in the main multi-center trial presented the greatest cognitive changes (Sink et al., 2015). Interestingly, no differences were found on any cognitive tests (RAVLT or Stroop) for the LIFE multi-component exercise intervention compared to health education control either at the pilot phase (Williamson et al., 2009) or in the main trial involving 1,635 sedentary older participants (Sink et al., 2015).

Last, the selective attrition from dance classes based on poorer performance on executive function tests, reflects self-selection of individuals who are fast and accurate responders who had less “room” for change at this “dose” of intervention. This was not the case in the walking group whose attrition was not dependent on cognitive ability. Hence, our power to detect a significant effect over walking, if it exists, was further reduced.

Despite longitudinal studies showing cognitive benefits of social engagement, the effect of exercise interventions or dance-based intervention on social networking of older adults has rarely been studied. Maki et al. (2012) also did not find an effect of increased social connections in a walking group intervention delivered to community-dwelling older Japanese using the Lubben tool, as we did. The lack of effect can be explained by of the low number of men partners and the fact that the women already had high social and family connections.

### Empirical Evidence Supporting Cognitive Benefits of Multi-Dimensional Physical Activity

To the best of our knowledge, this is the first study examining the long-term cognitive benefits of dance intervention employing a randomized-controlled design. We hypothesized that dance would elicit greater improvements in cognitive functions due to the involvement of several cognitive domains while dancing than a functional aerobic activity, such as walking, presumably of lower cognitive load, although cross sectional observation suggests that walking in open environment poses cognitive challenges as well (Prohaska et al., 2009). Of all the cognitive tests, dance participants performed better on visuospatial immediate and delayed recall. The most likely explanation is that spatial learning and memory is useful for learning dance, and that participants doing the dance intervention may have practiced this skill to help remember dance steps leading to improvement. To date, only five dance-based interventions to improve cognitive function have been conducted and reported in the literature (Coubard et al., 2011; Kim et al., 2011; Kimura and Hozumi, 2012; Kattenstroth et al., 2013; Eggenberger et al., 2015; Hackney et al., 2015). Kim et al. (2011) found significant improvement in word list delayed recall and word list recognition following twice weekly Latin dancing (the Cha–Cha) over 6 months among older volunteer participants who had never danced before, although spatial memory was not assessed. Kattenstroth et al. (2013) reported major improvement in non-verbal learning test of geometric items (from the Repeatable Battery of Neuropsychological Status) among older participants with no record of dancing or sporting activity for 5 years. By contrast, Hackney et al. (2015) found no improvements in cognitive tests following 3 months adapted tango program delivered to seniors aged 59–95.

Two other studies examined a similar hypothesis with Tai-Chi (Taylor-Piliae et al., 2010) and Yoga (Oken et al., 2006) interventions, both considered as multi-dimensional physical activities, but the results were conflicting; in a well-designed RCT by Oken et al. no differences were found in several tests assessing the attention domains (e.g., Stroop test, Wisconsin Card-Sorting) between older adults practicing Yoga, walking, or wait-listed control. As with our study, small improvements of similar magnitude were noted in all groups. By contrast, Taylor-Piliae et al. (2010) reported greater improvements in working memory tests for Tai-Chi participants compared to traditional exercise class or wait-listed control. Two other studies tested a slightly different hypothesis by increasing mental load during aerobic training such as virtual reality tours and competitions while cycling (Andreson-Hanley et al., 2012), walking on treadmill while practicing verbal memory or virtual video-dance game (Eggenberger et al., 2015). Both studies showed advantages of the combined mental and physical load, but only in specific domains such as shifting attention and working memory (Eggenberger et al., 2015), probably the domains that were specifically trained whilst playing the video games.

### Feasibility of Implementing Community-Based Dance Program Tailored to Older Adults

The challenges of conducting a pragmatic trial must be acknowledged. First, we set very specific exclusion criteria but had to reduce the lower age limit to 60 years to boost our recruitment. In Australia, the prevalence of dance participation is low with only 2.1% of older adults reported to dance yearly between 2000 and 2010 (Merom et al., 2012). Part of the reason is lack of infrastructure (teachers and studios) for dance classes catering to seniors. We were able to demonstrate an unmet demand in the DAnCE fall prevention trial by recruiting 15% of older residents in 23 retirement villages, of whom one-third had never tried dancing before (Merom et al., 2013). It was also difficult to find studio owners with experience of working with older populations; most community-based studios focused on younger age groups and professional ballroom dancing. Second, participants were heterogeneous in terms of learning and progression of the dances, which challenged instructors. This was also echoed by the dance participants; some stating that it was not challenging enough, whereas others may have found it too difficult and withdrew. Hence, future community-based programs must be tailored to different levels of skill (i.e., novice, and prior experience). On the other hand, those enrolled in the walking group preferred to walk in their own time; this is also typical of Australia, where only 3% of habitual walkers engaged in organized walks (Merom et al., 2012). Over the trial period the numbers who attended the park walk declined.

## Strengths and Limitations

This trial has significant methodological advances over previous dance-based trials, including randomization, concealment, repeated baseline measurements, *a priori* sample size calculations, longer exposure, and larger sample than all dance trials. However, several limitations must be acknowledged: we did not monitor compliance with 2-h walking. Also, we may have introduced greater heterogeneity between-groups by covering several dance styles from the ballroom repertoire, which reduced the standardization of the program across five communities and instructors. Providing variety might have also impacted on progression to greater challenge. Other dance studies, although short-term, have sticked to one specific style (i.e., Cha–Cha, tango, line dancing, Caribbean, Waltz). Measurements were conducted in different studios, which might have introduced greater random error due to different settings in terms of noise, type of floor surface, etc., and finally the study could have benefited from a third arm of a wait-listed control.

## Conclusion

The superior potential of dance over walking on executive functions of cognitively healthy and active older adults was not supported. Dance improved one of the cognitive domains (spatial memory) important for learning dance. It is possible that attrition from dance by those with poorer cognitive ability compromised our ability to detect a greater effect over walking; although, it may also indicate that dance poses cognitive challenges that not everyone can face unless the level is adapted to cognitive status. Hence, future RCTs should target inactive older adults and interventions of increased intensity and dose should be conducted, adapted to cognitive status, in order to produce stronger effects.

## Author Contributions

DM conceived the study and drafted the first manuscript. DM, AG, RE, and KA led the design of the work. JM led the field implementation, guided the interventions, and prepared the data for analyses. DM, AG, and BJ engaged in the analyses. DM, AG, RE, BJ, and KA contributed to the interpretation of the data. All authors worked on the revisions of the drafts, critically contributed to its content, and approved the final version of the manuscript.

## Conflict of Interest Statement

The authors declare that the research was conducted in the absence of any commercial or financial relationships that could be construed as a potential conflict of interest.
